# The impact of emotional inhibition trajectories on clinical pregnancy in infertile women undergoing assisted reproduction

**DOI:** 10.7717/peerj.21238

**Published:** 2026-05-11

**Authors:** Sihua Zhao, Rong Hu, Lihong Zhang

**Affiliations:** 1 Second Ward of Urology, The First Hospital of Lanzhou University, Lanzhou, China; 2 School of Nursing, Lanzhou University, Lanzhou, China

**Keywords:** Infertility, Emotional inhibition, Clinical pregnancy rate, Trajectory

## Abstract

**Background:**

Emotional inhibition can contribute to the development of anxiety and depression, which may adversely affect clinical pregnancy rate in infertile women. However, the impact of emotional inhibition trajectories on clinical pregnancy rates in this population remains unclear.

**Objective:**

This study aimed to investigate the impact of emotional inhibition trajectories on clinical pregnancy rate in infertile women undergoing assisted reproduction.

**Methods:**

This study was conducted at the Reproductive Center of a tertiary hospital in Lanzhou, Northwest China, from March 2024 to March 2025. A total of 655 participants were enrolled. Emotional inhibition was assessed at two critical time points to delineate its change trajectories (categorized as worsening, stable, or improvement). Clinical and laboratory data were collected, and participants’ clinical pregnancy rate were followed up. Binary logistic regression was used to analyze the association between emotional inhibition trajectories and clinical pregnancy.

**Results:**

Three distinct emotional inhibition trajectories were identified: the worsening group, the stable group, and the improvement group. Endometrial thickness on the day of embryo transfer, emotional inhibition trajectory, and the ovarian stimulation protocol were identified as significant factors associated with clinical pregnancy. Notably, compared with the improvement group, the worsening group was associated with significantly lower odds of achieving clinical pregnancy (OR = 0.144, 95% CI [0.087–0.239]).

**Conclusions:**

Infertile women with a worsening emotional inhibition trajectory had significantly poorer clinical pregnancy rate compared to those with an improvement trajectory. This suggests that progressive emotional inhibition may be a critical psychological risk factor for adverse pregnancy outcomes. These findings underscore the clinical importance of monitoring both the levels and dynamic changes of emotional inhibition, and they support implementing timely psychological interventions to potentially optimize clinical pregnancy rate.

## Introduction

The diagnostic and treatment process for infertility typically involves repeated cycles of hormonal medications, frequent venipunctures for blood tests, and multiple hospital visits. These repetitive and often invasive procedures can result in significant physical discomfort and psychological distress (Ni, Tong & Han, 2017). Such cumulative burdens may lead infertile women to develop or experience heightened negative emotions, such as anxiety, depression, anger, and sadness (He & Lei, 2014; Ramezanzadeh et al., 2004). When experiencing negative emotions, infertile women may consciously suppress their emotional expressions—a behavior known as emotional inhibition—often in an attempt to alleviate family concerns. This results in the internalization of distress and the presentation of a composed exterior. Emotional inhibition is defined as the conscious suppression of one’s emotional expression and the concealment of emotions following emotional arousal (Gross, 2002). Emotional inhibition is increasingly recognized as a common psychological phenomenon among infertile women. For instance, a study by Zheng et al. (2025) reported that a considerable proportion of women undergoing *in vitro* fertilization and embryo transfer exhibited moderate to high levels of emotional inhibition, with over 30% of participants scoring above the clinical threshold. This finding underscores the prevalence of this condition within this vulnerable population. Previous research has identified several factors associated with emotional inhibition in infertile women, including cultural expectations, lack of social support, and perceived stigma related to infertility (Hu, 2025). Although limited, intervention studies have suggested that expressive arts-based therapies, such as Mandala Therapy, may help reduce emotional inhibition and improve psychological well-being in medically ill patients (Ma, 2024). However, most existing studies have adopted cross-sectional designs, focusing on static associations rather than on the dynamic trajectories of emotional inhibition over time.

Prolonged emotional inhibition may exacerbate negative emotional states, placing the body in a sustained state of stress. Chronic stress can lead to reduced immune function and increase susceptibility to various diseases, which may ultimately diminish the likelihood of achieving pregnancy in infertile women (Li et al., 2024). Furthermore, emotional inhibition contributes to the development of anxiety and depression in this population, adversely affecting their quality of life, mental health, and overall well-being (Vaughan et al., 2019; Butler, Lee & Gross, 2007). It is well-established that psychological distress, such as anxiety and depression, is associated with reduced pregnancy rates in women undergoing infertility treatment (Turner et al., 2013; Frederiksen et al., 2015). Furthermore, given that emotional inhibition is a recognized risk factor for the development of such negative emotions (Gross, 2002), it may indirectly contribute to the lower clinical pregnancy rate observed in this population. Therefore, it is reasonable to posit that prolonged emotional inhibition may indirectly impair reproductive health by exacerbating anxiety and depression, thereby reducing the likelihood of successful conception. Despite increasing evidence linking emotional inhibition to adverse psychological outcomes, few studies have examined how changes in this condition over the course of fertility treatment influence pregnancy outcomes. Furthermore, the trajectory of emotional inhibition—whether it worsens, improves, or remains stable—has not been adequately investigated in relation to clinical pregnancy success. To address this gap, this prospective study aimed to examine the association between the dynamic trajectories of emotional inhibition during treatment and clinical pregnancy rate in infertile women undergoing assisted reproductive technology (ART).

## Material and methods

### Study sample

Formal participant recruitment and data collection commenced after obtaining ethical approval and were conducted from March 2024 to March 2025. Inclusion criteria were: (1) undergoing *in vitro* fertilization (IVF) or intracytoplasmic sperm injection (ICSI); (2) age ≤ 45 years; (3) ability to complete the self-assessment questionnaires voluntarily and independently; (4) moderate or higher scores on the initial emotional inhibition assessment (EI > 19); (5) availability for follow-up until clinical pregnancy confirmation. Exclusion criteria were: (1) congenital reproductive tract anomalies, history of tumors, history of pelvic or uterine surgery, or concurrent major systemic diseases; (2) voluntary withdrawal during the study; (3) incomplete questionnaire data; (4) current or recent diagnosis of major psychiatric disorders (*e.g.*, major depressive disorder, anxiety disorders). All participants provided written informed consent. This study was approved by the Ethics Committee of the School of Nursing, Lanzhou University (Approval No. LZUHLXY20240023) and was conducted in accordance with the ethical principles of the Declaration of Helsinki.

### Emotional inhibition scale

The Emotional Inhibition Scale (EIS), developed by Grandi et al. (2011), was used to assess an individual’s tendency to inhibit emotional expression. The scale comprises 14 items across four subscales: timidity, verbal inhibition, self-control, and emotional camouflage. Respondents rate each item on a 5-point Likert scale ranging from 0 (“cannot”) to 4 (“always”). The total score ranges from 0 to 56, with higher scores indicating a greater level of emotional inhibition. The EIS has demonstrated good reliability and validity in previous clinical studies (Zheng et al., 2025). The full scale is provided in the Supplemental Information.

### Data collection and measures

This study employed a prospective design. The level of emotional inhibition was assessed using the EIS at two critical time points: (1) the day of human chorionic gonadotropin (hCG) injection (T1), and (2) the day of embryo transfer (T2). Based on the score change between T1 and T2, participants were categorized into three trajectory groups: the worsening group (an increase of > 4 points), the improvement group (a decrease of > 4 points), and the stable group (a change within ±4 points).

Clinical and laboratory data were extracted from electronic medical records. Demographic and historical factors included age, duration of infertility, body mass index, and history of adverse maternal outcomes. Treatment cycle parameters consisted of the ovarian stimulation protocol, fertilization method, total gonadotropin dose, number of oocytes retrieved, number of embryos transferred, stage of embryo transfer, and endometrial thickness on the day of transfer. Hormonal profiles encompassed basal follicle-stimulating hormone level, anti-Müllerian hormone level, and serum levels of estradiol and progesterone on the day of hCG injection. The primary outcome was clinical pregnancy, operationally defined as the ultrasound confirmation of an intrauterine gestational sac with cardiac activity at 4 to 5 weeks after embryo transfer. An initial urine pregnancy test was performed on day 14 post-transfer as a screen for biochemical pregnancy

### Statistical methods

In this study, data entry was performed using Excel. Statistical analyses were conducted with SPSS 29.0 (IBM Corp., Armonk, NY, USA) and AMOS 26.0 software after data cleaning. A two-sided test was applied, and a *P*-value of <0.05 was considered statistically significant.

(1) Descriptive Statistics: Categorical variables were summarized using frequencies and percentages. Continuous variables were summarized using means and standard deviations if normally distributed, or medians and interquartile ranges if non-normally distributed.

(2) Univariate Analysis: Variables were compared across groups using parametric tests (*t*-test, ANOVA) or non-parametric tests (Chi-square test, Kruskal-Wallis H test) as appropriate, based on data type and distribution.

(3) Binary Logistic Regression Analysis: multivariable logistic regression analysis was performed to identify independent factors associated with clinical pregnancy rate. Clinical pregnancy (yes/no) served as the dependent variable. Independent variables included in the final model were those showing a statistically significant association (*P* < 0.05) with the clinical pregnancy rate in the univariate analyses. This approach was adopted to construct a parsimonious model that adjusts for these significant covariates while evaluating the independent association of the emotional inhibition trajectory with clinical pregnancy rate.

## Results

**Figure 1:**
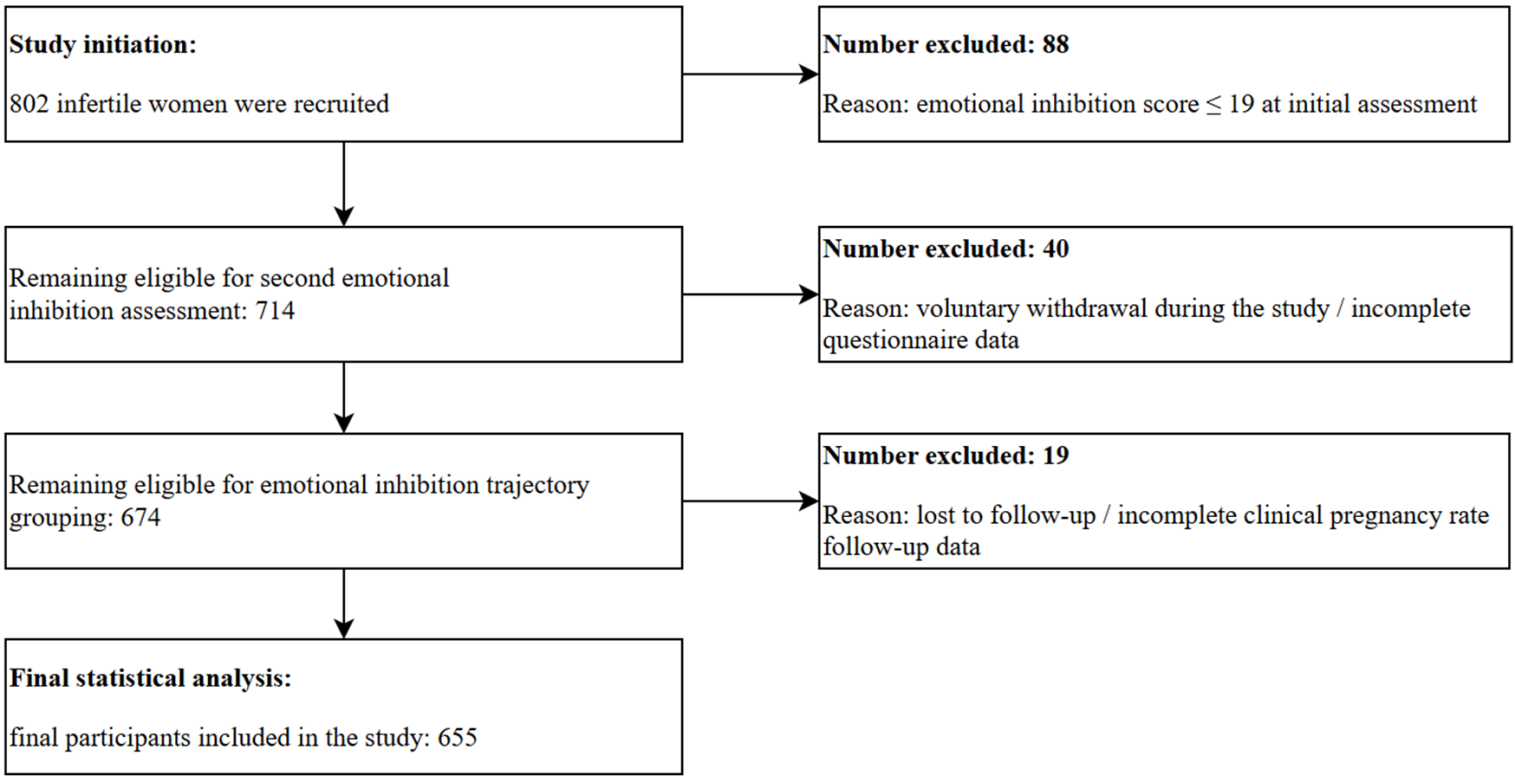
Flow diagram of participant recruitment, screening, and enrollment.

Between March 2024 and March 2025, this study prospectively recruited 802 infertile women. The participant screening process was as follows: after the first emotional inhibition assessment, 88 participants with scores ≤19 were excluded, leaving 714 participants. After the second assessment, a further 40 participants were excluded (due to withdrawal or incomplete data), resulting in 674 participants for trajectory grouping. Ultimately, 655 participants were successfully followed up with clinical pregnancy data available and were included in the final analysis. See Fig. 1 for participant recruitment flow.

### Baseline characteristics of the study participants

The baseline characteristics of the 655 participants are summarized in Table 1. The cohort had a mean age of 32.0 years and a mean body mass index (BMI) of 22.9 kg/m^2^. The majority underwent a gonadotropin-releasing hormone agonist (GnRH-a) protocol for ovarian stimulation (*n* = 500, 76.3%) and IVF (*n* = 461, 70.4%).

**Table 1:** Baseline characteristics of the study sample and emotional inhibition trajectory groups.

**Characteristic**	Worsening group (*n* = 374)	Stable group (*n* = 176)	Improvement group (*n* = 105)	Total (*N* = 655)
Age (years)	31.98 ± 4.02	32.10 ± 4.60	31.97 ± 3.69	32.0 ± 4.12
Duration of infertility (years)	3.89 ± 2.63	3.61 ± 2.19	3.88 ± 3.17	3.81 ± 2.61
Body mass index (kg/m^2^)	22.77 ± 3.35	23.13 ± 3.17	22.95 ± 2.92	22.90 ± 3.23
**Ovarian stimulation protocol**				
GnRH-a	277 (42.3)	139 (21.2)	84 (12.8)	500 (76.3)
GnRH-ant	97 (14.8)	37 (5.6)	21 (3.2)	155 (23.7)
**Fertilization method**				
IVF	270 (41.2)	120 (18.3)	71 (10.8)	461 (70.38)
ICSI	104 (15.9)	56 (8.5)	34 (5.2)	194 (29.62)
**Stage of embryo transfer**				
Cleavage-stage embryo	211 (32.2)	89 (13.6)	61 (9.3)	361 (55.11)
Blastocyst-stage embryo	163 (24.9)	87 (13.3)	44 (6.7)	294 (44.89)
**Previous adverse pregnancy history**				
No	224 (34.2)	91 (13.9)	56 (85.5)	371 (56.64)
Yes	150 (22.9)	85 (13.0)	49 (7.5)	284 (43.36)
Endometrial thickness on the day of embryo transfer (mm)	11.10 ± 2.00	11.30 ± 2.01	11.25 ± 2.12	3.56 ± 3.08
Anti-Müllerian Hormone (AMH) level (ng/mL)	3.48 ± 2.85	3.65 ± 3.12	3.70 ± 3.73	11.18 ± 2.03

**Notes.**

x ± s/n, (%).

### Emotional inhibition in infertile women

In this study, the emotional inhibition scores at T1 ranged from 19 to 53, with a mean (±SD) of 28.42 ± 6.34. The scores at T2 ranged from 14 to 50, with a mean of 30.58 ± 5.42. Based on the score changes between the two assessments, participants were categorized into three trajectories: the worsening group (*n* = 374, 57.1%), the stable group (*n* = 176, 26.9%), and the improvement group (*n* = 105, 16.0%). The emotional inhibition scores at both time points for each trajectory group are presented in Table 2.

**Table 2:** Emotional inhibition scores.

	Total EI *N* (%)	Worsening group 374 (57.1%)	Stable group 176 (26.9%)	Improvement group 105 (16.0%)
T1	28.42 ± 6.34	26.3 ± 4.81	28.85 ± 5.77	36.31 ± 6.07
T2	30.58 ± 5.42	31.24 ± 4.95	29.64 ± 5.74	29.8 ± 6.10

### Univariate analysis

Normality tests indicated that all continuous variables in this study approximately followed a normal distribution; therefore, parametric tests (*t*-tests or ANOVA) were employed for group comparisons. Categorical variables were analyzed using the Chi-square test. The univariate analysis results are presented in Table 3. Five factors demonstrated a statistically significant association with clinical pregnancy: age, endometrial thickness on the day of embryo transfer, BMI, emotional inhibition trajectory, and the ovarian stimulation protocol.

**Table 3:** Univariate analysis of factors associated with clinical pregnancy.

**Variable**	**Clinical pregnancy group** **(*n* = 318)**	**Non-** **clinical pregnancy group (*n* = 337)**	***t*** **/*χ*** ^**2**^	***P***
Age (years)	31.83 ± 3.90	32.18 ± 4.32	−1.58	0.048
Number of oocytes retrieved	14.30 ± 6.15	13.74 ± 6.78	1.10	0.271
Number of embryos transferred	1.62 ± 0.49	1.56 ± 0.49	1.51	0.131
Infertility duration (years)	3.92 ± 2.74	3.71 ± 2.50	0.99	0.323
Day of embryo transfer (mm)	11.39 ± 1.91	10.97 ± 2.11	2.70	0.007
BMI (Kg/m^2^)	23.20 ± 3.35	22.61 ± 3.10	2.31	0.021
AMH (ng/mL)	3.62 ± 3.23	3.50 ± 2.93	0.49	0.618
FSH (U/L)	7.24 ± 4.13	7.18 ± 4.05	0.17	0.862
E2 (pg/mL)	3,685.19 ± 1,860.82	3,734.32 ± 1,834.87	−0.34	0.734
Total Gonadotropin Dose (U)	2,219.34 ± 678.98	2,198.45 ± 764.67	0.73	0.440
P (ng/mL)	1.05 ± 0.73	1.14 ± 0.63	−1.78	0.074
**Emotional inhibition trajectory**			102.53	<0.001
Worsening group	118 (18.0)	256 (39.1)		
Stable group	120 (18.3)	56 (8.6)		
Improvement group	80 (12.2)	25 (3.8)		
**Ovarian stimulation protocol**			8.94	0.003
GnRH-a	259 (39.54)	241 (36.79)		
GnRH-ant	59 (9.0)	96 (14.7)		
**Fertilization method**			0.01	0.975
IVF	224 (34.2)	237 (36.2)		
ICSI	94 (14.4)	100 (15.3)		
**Stage of embryo transfer**			0.97	0.325
Cleavage-stage embryo	169 (25.8)	192 (29.3)		
Blastocyst-stage embryo	149 (22.8)	145 (22.1)		
**Previous adverse pregnancy history**			0.42	0.516
No	176 (26.9)	195 (29.8)		
Yes	142 (21.7)	142 (21.7)		

### Logistic regression analysis

**Figure 2:**
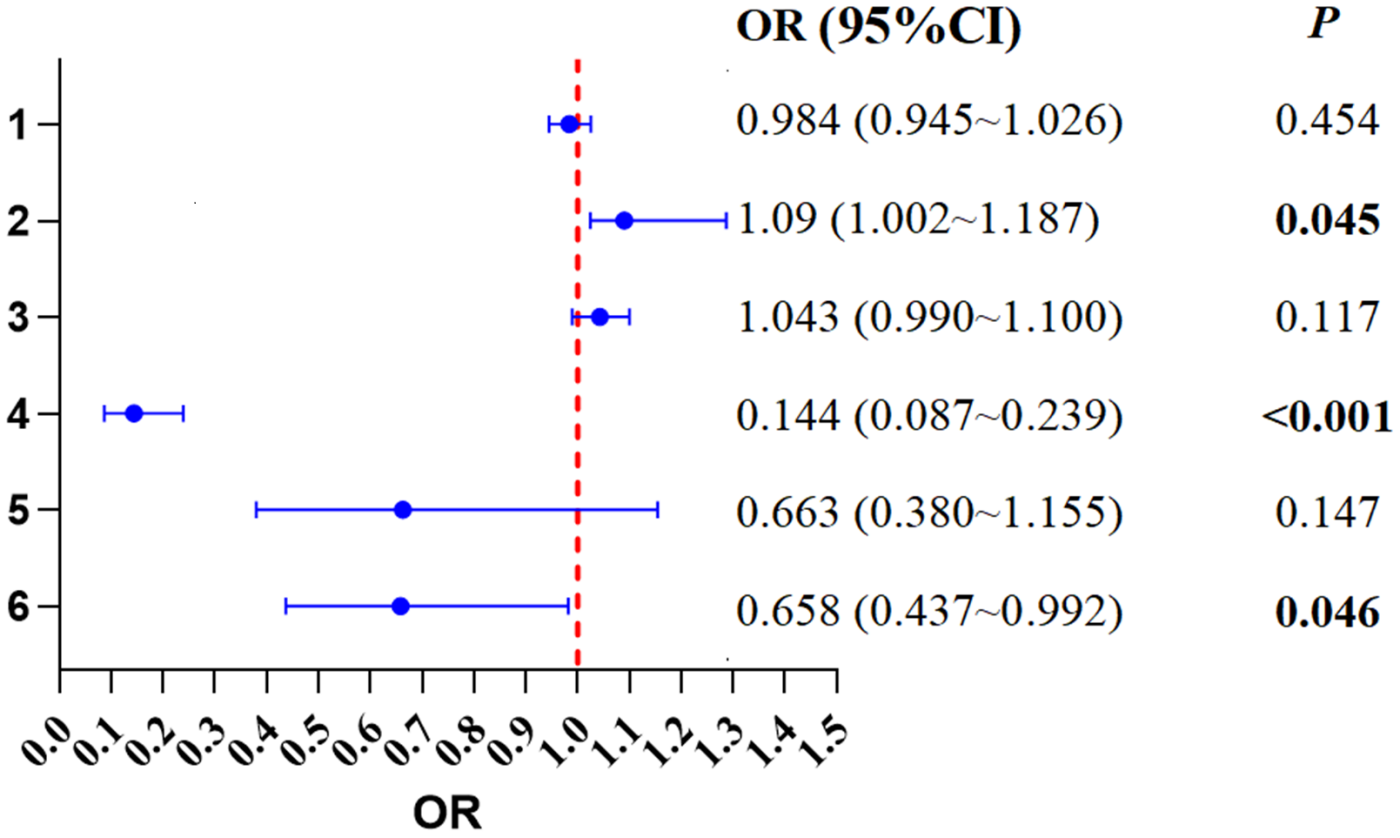
Logistic regression analysis of the impact of emotion suppression trajectories on pregnancy outcomes.

The multivariable logistic regression model demonstrated good fit to the data, as indicated by a non-significant Hosmer-Lemeshow test result (*χ*^2^ = 4.74, *P* = 0.656). The model explained 21.9% of the variance in clinical pregnancy (Nagelkerke *R*^2^ = 0.219). The analysis identified endometrial thickness on the day of embryo transfer, emotional inhibition trajectory, and the ovarian stimulation protocol as significant independent factors. Specifically, each one mm increase in endometrial thickness was associated with a 9% increase in the odds of clinical pregnancy (OR = 1.09, 95% CI [1.002–1.187]). In contrast, women in the worsening emotional inhibition trajectory group had 85.6% lower odds of achieving clinical pregnancy compared to those in the improvement group (OR = 0.144, 95% CI [0.087–0.239]). Furthermore, the use of a Gonadotropin-Releasing Hormone antagonist (GnRH-ant) protocol was associated with a 34.2% reduction in the odds of pregnancy compared to the GnRH-a protocol (OR = 0.658, 95% CI [0.437–0.992]). See Fig. 2 for details.

## Discussion

This study included a total of 655 infertile women. The overall level of emotional inhibition in the cohort was moderate. Among them, 81 participants (12.4%) exhibited a high level of emotional inhibition, a finding that is consistent with previous reports by Zheng et al. (2025). However, it should be noted that reported levels of emotional inhibition vary considerably across studies, likely due to differences in study populations, assessment instruments, and research contexts. Li et al. (2015) investigated emotional inhibition in a breast cancer population and reported scores at a medium-high level, which were slightly higher than those observed in the present study of infertile women. Similarly, Zhu et al. (2025) found that 32.3% of gastric cancer patients exhibited a high level of emotional inhibition, a proportion exceeding that in our cohort. These discrepancies may be attributable to differences in disease severity. As cancer is a life-threatening condition, patients often experience persistent fear and distress, which may generally lead to higher levels of emotional inhibition.

This study constructed emotional inhibition trajectories based on scores from two assessments, aiming to explore the impact of dynamic changes in emotional inhibition on clinical pregnancy among infertile women. The results demonstrated that a worsening emotional inhibition trajectory is a significant risk factor for achieving clinical pregnancy (OR = 0.144, 95% CI [0.087–0.239]). Emotional inhibition is defined as a behavioral response in which an individual consciously suppresses emotional expression following emotional arousal triggered by stressors such as life events or adverse experiences (Shen, Hu & Wang, 2011). In this study, emotional inhibition was assessed using the EIS, which measures dimensions including emotional camouflage, self-control, verbal inhibition, and timidity.

Emotional inhibition can be conceptualized as the suppression of one’s own emotions without adequate release. This sustained suppression may act as an invisible barrier to successful pregnancy. Research indicates that emotional inhibition can exacerbate initial negative emotions, leading to a greater variety and intensity of such emotions over time (Vaughan et al., 2019; Peterson et al., 2006). The accumulation of these negative emotions can adversely affect women’s physiological functions, labor and delivery processes, clinical pregnancy, and future neonatal health, thereby increasing the difficulty for infertile women to achieve a clinical pregnancy (Li et al., 2024). Studies have indicated that a tendency toward emotional inhibition can significantly exacerbate the pain and psychological distress experienced by women with primary infertility (Cesta et al., 2016; Hoyle, Davisson & Novice, 2022). Furthermore, it contributes to elevated psychological stress and fosters the development of serious psychological disorders, such as anxiety and depression, in infertile women (Bayley, Slade & Lashen, 2009). This increase in psychological stress and affective disorders may, in turn, adversely affect pregnancy outcomes (Cui et al., 2020). The long-term inhibition of emotions without appropriate outlets can facilitate the generation of negative emotions, placing the body in a chronic state of stress. Sustained stress may lead to reduced immune function and increased susceptibility to various diseases, ultimately diminishing the likelihood of achieving pregnancy in infertile women (Li et al., 2024). Furthermore, emotional inhibition is closely linked to an individual’s internal traits and is not easily modified in the short term. If an infertile patient habitually suppresses and conceals her emotions, even after achieving pregnancy and during its early stages, this pattern may adversely affect fetal development (Belda et al., 2015; Yu & Yang, 2013).

Previous studies have established that emotional inhibition contributes to the development of anxiety and depression, both of which are recognized risk factors for adverse pregnancy outcomes in infertile women. Therefore, the findings of this study suggest that healthcare professionals should consider implementing early interventions to address emotional inhibition in this population. Timely management may serve the dual purpose of preventing the onset or escalation of anxiety and depression, and of potentially improving the likelihood of successful pregnancy.

Our multivariable model underscores that pregnancy outcome is determined by a confluence of psychological, endometrial, and iatrogenic factors. Consistent with extensive literature, endometrial thickness on the day of embryo transfer emerged as a significant positive predictor of clinical pregnancy (Wang et al., 2025). A thicker endometrium typically reflects adequate estrogen priming and enhanced receptivity, thereby providing a more favorable environment for embryo implantation (Luo et al., 2021). Our finding that each 1-mm increase in endometrial thickness was associated with a 9% increase in the odds of clinical pregnancy reinforces the critical role of endometrial receptivity as a fundamental prerequisite for success in ART.

Furthermore, the ovarian stimulation protocol emerged as an independent factor. The use of a GnRH-ant protocol was associated with a 34% reduction in the odds of pregnancy compared to the GnRH-a protocol. This finding aligns with reports suggesting that while GnRH-ant protocols offer advantages such as shorter treatment duration and a lower risk of severe ovarian hyperstimulation syndrome (OHSS), they may be associated with a marginally lower pregnancy rate in certain populations. This difference could be attributable to variations in endometrial receptivity or oocyte quality (Yang et al., 2021). Consequently, these results underscore the importance of individualized treatment planning, wherein the choice of stimulation protocol should be carefully tailored to the patient’s specific clinical profile.

## Limitation

This study has several limitations. First, it was conducted at a single center, which may limit the generalizability of the findings to other populations or clinical settings. Second, the assessment of emotional inhibition relied solely on self-report measures, which are susceptible to biases such as social desirability. Future studies could incorporate objective physiological indicators to complement self-report data. Third, the primary outcome was clinical pregnancy; live birth data were not available due to the study’s timeframe and the logistical challenge of obtaining complete follow-up information after the principal investigator’s graduation. Fourth, although the interval between the two emotional inhibition assessments (T1 and T2) was clinically standardized for most participants, its potential confounding effect was not formally analyzed. Finally, although the logistic regression model was statistically significant, the proportion of variance explained (Nagelkerke *R*^2^ = 0.219) was modest, suggesting that other unmeasured factors also substantially influence clinical pregnancy.

## Conclusions

In infertile women, a worsening trajectory of emotional inhibition was associated with significantly poorer clinical pregnancy rate compared to an improvement trajectory. This finding suggests that progressively worsening emotional inhibition is an important psychological risk factor for a poorer clinical pregnancy rate. Therefore, clinical attention should focus on monitoring the dynamic changes in emotional inhibition, with timely psychological support offered to individuals exhibiting a deteriorating pattern to potentially optimize treatment success. Future research should investigate whether the impact of these trajectories varies across different baseline levels of emotional inhibition, which could inform the development of stratified psychological interventions.

## Supplemental Information

10.7717/peerj.21238/supp-1 Supplemental Information 1 Emotional Inhibition Scale

10.7717/peerj.21238/supp-2 Supplemental Information 2 Raw data

10.7717/peerj.21238/supp-3 Supplemental Information 3 STROBE Checklist

## Additional Information and Declarations

### Data Availability

The following information was supplied regarding data availability: The raw data is available in the Supplemental File.
