# Supplementary material for: The impact of emotional inhibition trajectories on clinical pregnancy in infertile women undergoing assisted reproduction
Source: PeerJ. 2026 May 11;14:e21238. doi: 10.7717/peerj.21238 (PMC13175059; doi:10.7717/peerj.21238)
Supplement: Supplemental Information 1 [file peerj-14-21238-s001.docx]

**Emotional Inhibition Scale**

Please read all questions carefully. Each question has five options. Choose the one that best describes your situation.

1. Even if someone is rude to you, do you still treat them politely?

□ Never □ Seldom □ Sometimes □ Most of the time □ Always

1. Do you find it easy to talk to others about your feelings?

□ Never □ Seldom □ Sometimes □ Most of the time □ Always

1. When you feel misunderstood, do you find it difficult to defend yourself?

□ Never □ Seldom □ Sometimes □ Most of the time □ Always

1. Do you express your feelings?

□ Never □ Seldom □ Sometimes □ Most of the time □ Always

1. Do you tell others your true thoughts?

□ Never □ Seldom □ Sometimes □ Most of the time □ Always

1. Do you find it difficult to stand up for your rights?

□ Never □ Seldom □ Sometimes □ Most of the time □ Always

1. Even with close friends, do you find it hard to talk about your true feelings?

□ Never □ Seldom □ Sometimes □ Most of the time □ Always

1. When you are angry, do you try to control your emotions?

□ Never □ Seldom □ Sometimes □ Most of the time □ Always

1. When you feel anxious or worried, do you try to appear calm?

□ Never □ Seldom □ Sometimes □ Most of the time □ Always

1. Do you refrain from saying something because it might hurt others?

□ Never □ Seldom □ Sometimes □ Most of the time □ Always

1. Do you ever feel that others are taking advantage of you?

□ Never □ Seldom □ Sometimes □ Most of the time □ Always

1. Even when you feel sad, do you pretend to be happy?

□ Never □ Seldom □ Sometimes □ Most of the time □ Always

1. Have you ever wanted to tell others how you feel but finally didn’t because you felt embarrassed?

□ Never □ Seldom □ Sometimes □ Most of the time □ Always

1. Do you let your friends know how you are feeling?

□ Never □ Seldom □ Sometimes □ Most of the time □ Always
